# Design and Fabrication of Full Wheatstone-Bridge-Based Angular GMR Sensors

**DOI:** 10.3390/s18061832

**Published:** 2018-06-05

**Authors:** Shaohua Yan, Zhiqiang Cao, Zongxia Guo, Zhenyi Zheng, Anni Cao, Yue Qi, Qunwen Leng, Weisheng Zhao

**Affiliations:** 1 Fert Beijing Institute, BDBC, School of Electronic and Information Engineering, Beihang University, Beijing 100191, China; yanshaohua@buaa.edu.cn (S.Y.); zhenyizheng@buaa.edu.cn (Z.Z.); caoan@buaa.edu.cn (A.C.); 2 Beihang-Goertek Joint Microelectronics Institute, Qingdao Research Institute, Beihang University, Qingdao 266000, China; zhiqiangcao@buaa.edu.cn (Z.C.); gzx0106@buaa.edu.cn (Z.G.); 3 State Key Laboratory of Virtual Reality Technology and Systems, Beihang University, Beijing 100191, China; qy@buaa.edu.cn; 4 Goertek Inc., Weifang 261031, China

**Keywords:** GMR effect, angular sensor, full Wheatstone bridge, synthetic antiferromagnet

## Abstract

Since the discovery of the giant magnetoresistive (GMR) effect, GMR sensors have gained much attention in last decades due to their high sensitivity, small size, and low cost. The full Wheatstone-bridge-based GMR sensor is most useful in terms of the application point of view. However, its manufacturing process is usually complex. In this paper, we present an efficient and concise approach to fabricate a full Wheatstone-bridge-based angular GMR sensor by depositing one GMR film stack, utilizing simple patterned processes, and a concise post-annealing procedure based on a special layout. The angular GMR sensor is of good linear performance and achieves a sensitivity of 0.112 mV/V/Oe at the annealing temperature of 260 °C in the magnetic field range from −50 to +50 Oe. This work provides a design and method for GMR-sensor manufacturing that is easy for implementation and suitable for mass production.

## 1. Introduction

Giant magnetoresistive (GMR)-based magnetic sensors have attracted much attention since the GMR effect was discovered by Albert Fert and Peter Grünberg in the 1980s [1,2]. Compared to their counterparts anisotropic magnetoresistance (AMR) or Hall sensors, GMR sensors display greater sensitivity and lager linear range [3] and have wide applications in compasses, electrical current measurement, detection of biologic antibodies labeled by magnetic beads, or nondestructive testing [4,5,6,7,8]. A special type of GMR structure is the spin valve, which was proposed by Dieny [9] and consists of two ferromagnetic layers separated by a nonmagnetic conductor spacer, usually made of Cu. One of these layers is pinned by an antiferromagnetic layer such as IrMn or PtMn, while the other is free to rotate. The pinned layer could be replaced by a synthetic antiferromagnetic (SAF) structure to diminish the mutual magnetostatic coupling between the free and pinned layer [10,11].

**Figure 1:**
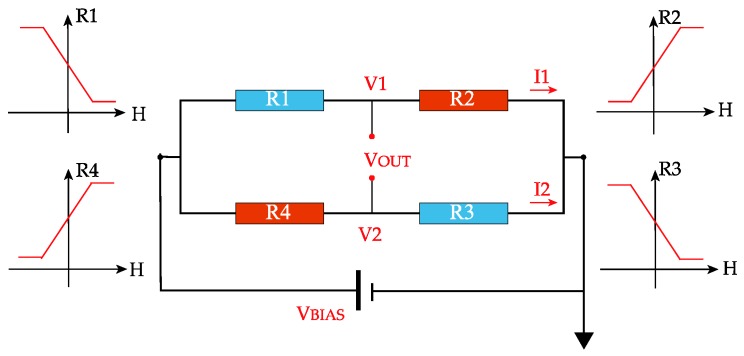
Schematic of a full bridge GMR sensor. It exhibits a null output in the absence of a signal field and offers an intrinsic compensation for thermal drift. When an external field H is applied, the bridge has a linear output. R_1,3_(H) = R − △R(H); R_2,4_(H) = R + △R(H); V_out_ = V_bias_*∆R/R.

For sensor application, a linear output is required, the easy axis of the pinned layer is usually arranged in the transverse orientation of a patterned stripe, and the easy axis of the free layer is in the longitudinal orientation [12]. In order to avoid output fluctuation caused by temperature drift, a Wheatstone bridge configuration is needed. However, if each of the MR elements has the same dimension and magnetization direction, each arm in the bridge responds identically to an applied field and the bridge generates no differential output. To make the bridge operational, a full Wheatstone bridge (see Figure 1) is very useful, in which two of bridge arms exhibit dR/dH > 0, while the remaining two exhibit dR/dH < 0 [13].

Several methods have been reported for the realization of a full bridge sensor. The simplest solution is to mechanically assemble GMR elements along the same direction but in opposite senses, which has important drawbacks such as alignment errors and is not suitable for mass production [13]. Another way is to deposit two stacks on different regions of the same wafer, and the magnetic fields used for two depositions are antiparallel. This is also a sophisticated process. Alternatively, after the fabrication of the bridge sensor, a local magnetic field annealing over each bridge element is done by a current flowing through the overlaying setting circuit to realign the magnetization of pinned layer [12,14]. The current flows in opposite directions for two pairs of resistors in the bridge. The amplitude of the current should be carefully calculated and a particular PCB is needed. A similar way is to use laser radiation in an applied field in place of the setting circuit [15,16]. However, all these methods are more or less complex.

In the present work, we demonstrated a concise experimental method for the realization of a full bridge GMR sensor using one GMR film stack. On the basis of a special layout, only a one-time post annealing procedure is required. In the following paragraphs, the details of this method as well as the characterization results are described.

## 2. Materials and Processes

**Figure 2:**
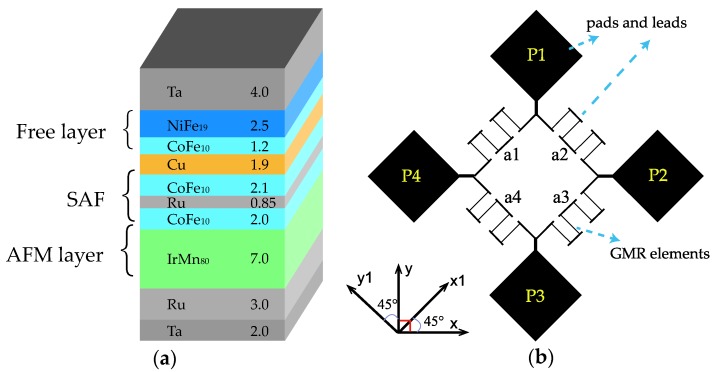
(**a**) Structure of GMR stack with SAF CoFe_10_/Ru/CoFe_10_. (**b**) Layout of Wheatstone bridge sensor. P1–P4 are four electrodes of the bridge used for electrical measurements. Each bridge arm (a1–a4) consists of four GMR stripes with dimensions of 3 × 225 μm connected in series. The meander connection of four stripes is used to obtain the resistance as designed in the bridge.

A thin film stack with a structure of 2.0 Ta/3.0 Ru/7.0 IrMn_80_/2.0 CoFe_10_/0.85 Ru/2.1 CoFe_10_/1.9 Cu/1.2 CoFe_10_/2.5 NiFe_19_/4.0 Ta (numbers denote layer thickness in nm) was deposited on SiO_2_ substrate with a base pressure less than 10^−8^ Torr by a Singulus ROTARIS ultra-high vacuum magnetron sputtering system. The CoFe_10_/Ru/CoFe_10_ part constituted a synthetic antiferromagnetic structure (see Figure 2a). The layout of the bridge sensor is illustrated in Figure 2b. The microelectronic fabrication process of the devices consisted of three lithographic steps. In the first one, the contact leads and pads were defined, using 10 nm Cr/80 nm Au by E-beam evaporation. Then, the GMR stripes were patterned by photolithography followed by ion-beam etching into active sensing elements with dimensions of 3 × 225 μm. The final passivation layer consisted of 80 nm SiO_2_ film by E-beam evaporation. The device was annealed in a vacuum at 250 °C under a negative field of 0.9 T along the *x*-axis for 1 h. Then, the field was changed to positive 50 mT for 15 min before cooling down without the field applied. Note that there was an angle of 45 degrees between the annealing direction and the longitudinal orientation of the GMR stripes. The R-H loops of the bridge arms and voltage output of the bridge were measured by a standard four-probe method at room temperature.

## 3. Results and Discussion

**Figure 3:**
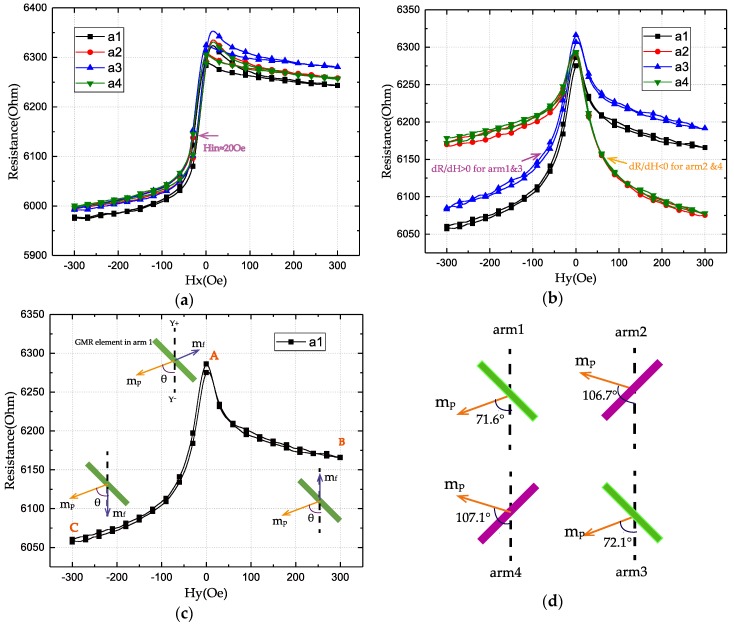
(**a**,**b**) R-H loops of the four bridge arms with the applied magnetic field along the *x*- and *y*-axes. (**c**) R-H loop of arm1 with the applied field along the *y*-axis. m_f_ and m_p_ denote the magnetization of the free and pinned layers. (**d**) Calculated deflection angles of pinned layer magnetization in GMR elements.

The R-H loops of each bridge arm are measured separately. A 1 μA DC current was used while applying an external field in different directions. The field utilized was swept from −300 Oe to +300 Oe and back to −300 Oe by a step of 6 Oe. Figure 3a illustrates that after annealing, the four arms of the bridge behave almost the same to the external field along the *x*-axis, where the output of the bridge should be null. The observed GMR ratio is 5.25%. It should be noted that the maximal intrinsic GMR ratio will be larger because the magnetization directions between the free and pinned layers are not antiparallel and parallel within the applied field. As the R-H loops have an offset from zero and enter the state of high resistance before H_x_ = 0 Oe, the magnetization of the free layer CoFe_10_/NiFe_19_ is antiferromagnetically coupled with the pinned layer across the spacer layer Cu. The coupling field H_in_ is about -20 Oe. For sensor application, a weak or no interlayer coupling is desired and it could be adjusted by tuning the layer thicknesses [17].

Figure 3b shows that arm1 and arm3 exhibit dR/dH > 0, while arm2 and arm4 exhibit dR/dH < 0. This conforms to the characteristics of a full bridge sensor along the *y*-axis. The resistance of the GMR element depends on the angle α between the free and pinned layers magnetization [18], described by

(1) $$R(\alpha )=R_{p}+(R_{ap}-R_{p})(1-\cos\alpha )/2$$

where *R_p_* and *R_ap_* are the resistances of parallel and antiparallel alignments of the free and pinned layers magnetization in the GMR element. The variation of resistance results from the rotation of free layer magnetization.

The R-H loops in Figure 3b are not symmetric around the *y*-axis, so the pinning direction is not perpendicular to the *y*-axis. We note the angle between pinned layer easy axis and *y*-axis as θ. To estimate θ, we suppose that our device stays in the single-domain state and the magnetic reversal occurs by coherent rotation of magnetization. At point A in Figure 3c, the external field H = 0 Oe. Due to the antiferromagnetic coupling between the free and pinned layer, their magnetizations are antiparallel, and the element has the highest resistance. At points B and C, the free layer magnetization follows the direction of the applied field. Using Equation (1), we obtain

(2) $$\frac{R_{A}-R_{B}}{R_{A}-R_{C}}=\frac{1+\cos(\pi -\theta )}{1+\cos\theta }=\frac{\cos^{2}(\frac{\pi -\theta }{2})}{\cos^{2}(\frac{\theta }{2})}=\tan^{2}(\frac{\theta }{2})$$

Then the angle *θ* can be expressed as

(3) $$\theta =2\arctan\sqrt{\frac{R_{A}-R_{B}}{R_{A}-R_{C}}}$$

The calculated result of *θ* for arm1 in the bridge is 71.6°. Same calculations are performed for the other arms, and these angles are 106.7°, 72.1°, and 107.1°, respectively, as shown in Figure 3d.

**Figure 4:**
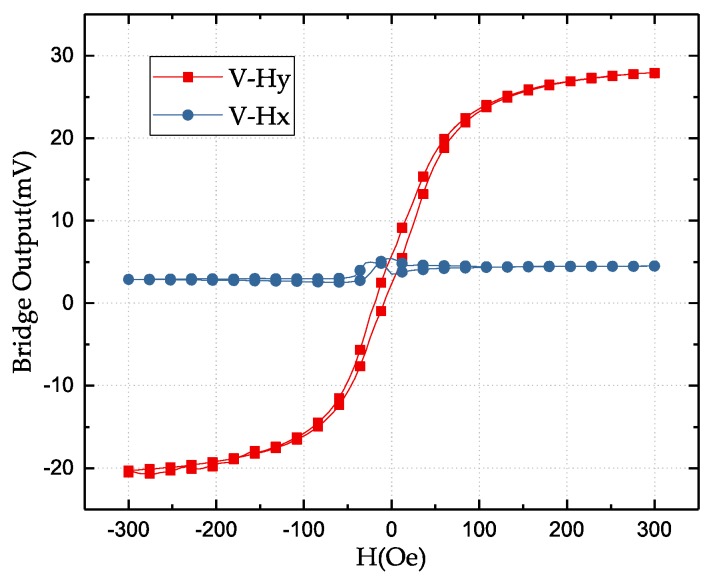
The output of the full Wheatstone-bridge-based GMR sensors under 3 V bias voltage in the x- and *y*-axes.

**Figure 5:**
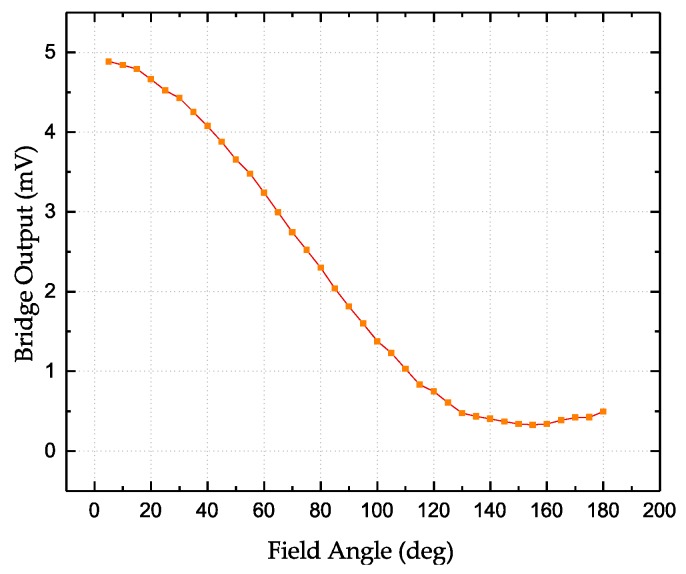
Bridge output as a function of the applied field direction.

Figure 4 shows the output of the full Wheatstone-bridge-based angular GMR sensor under bias voltage of 3 V. The angular sensor is designed to be only sensitive to the external field in the *y*-axis, i.e., the *y*-axis is the sensing direction. The response in the *x*-axis is almost a constant. The voltage offset is due to the different resistances and pinning directions of four resistors in the bridge. The sensitivity of this sensor measures 0.09 mV/V/Oe in the sensing axis. The bridge output as a function of the applied field angle is shown in Figure 5, where the magnitude of the field is fixed as 5Oe. A unique angle can only be determined between 0 and 180 degrees.

**Figure 6:**
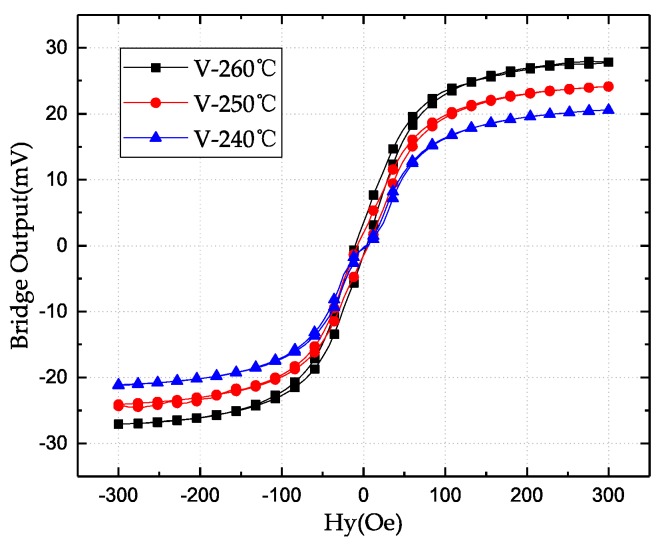
The outputs in the *y*-axis for the full Wheatstone-bridge-based GMR sensors annealed at different temperatures.

The outputs of the full bridge-based sensors in the sensing axis at different annealing temperatures are demonstrated in the Figure 6. The performances of the full bridge sensors annealed at different temperatures are listed in Table 1 for comparison. The magnetothermal process will change the sensor sensitivities that show an increase with the increases of the annealing temperature monotonically. The angle between the pinned magnetization and the *y*-axis should be 45° and 135° for bridge arms 1, 3 and 2, 4, respectively. The more the magnetization of the pinned layer is away from these angles, the less the sensitivity of the bridge sensor is. The GMR ratio is improved at higher annealing temperature, and the sensitivity is as well. It can be seen that the linearity of the bridge response is better at the higher annealing temperature, as indicated in Figure 6.

**Figure 7:**
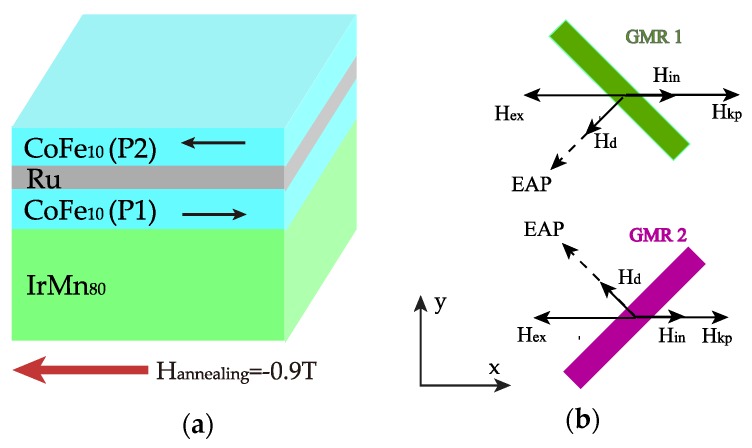
(**a**) Schematic of the exchange coupling field in SAF structure. (**b**) Direction of the pinned layer CoFe_10_(P2) easy axis (EAP) after annealing.

During the annealing process, the exchange coupling between IrMn and CoFe(P1) disappears when the temperature is higher than the blocking temperature of IrMn. With a field of −0.9 T applied in the *x*-axis, the exchange coupling field direction of IrMn/CoFe(P1) is set. After the field is removed, due to strong antiferromagnetic coupling in SAF and IrMn/CoFe, the magnetization of CoFe(P2) tends to align itself antiparallel to that of CoFe(P1), as indicated in Figure 7a. The annealing under the field of 50 mT will barely impact the direction of the exchange coupling field because the magnetic field threshold for spin flop in SAF is about 100 mT [18,19].

As shown in Figure 7b, in the GMR element 1, the easy axis of its pinned layer CoFe(P2) (EAP) is defined by the competition among the demagnetizing field H_d_, the exchange coupling field H_ex_ from SAF, the interlayer coupling field from free layer H_in_, and the induced anisotropy field H_kp_ [20]. Here, the short-time low-field annealing contributes to the induced anisotropy, taking effect to compensate the deviation of the easy axis direction. The ideal direction is along the transverse orientation of the patterned stripe.

The easy axis of the free layer is determined by the small interlayer coupling field H_in_, the induced anisotropy field H_kf_, and the demagnetizing field H_d_, which make it tend to align along the longitudinal orientation of the patterned stripe [21].

For a IrMn- or PtMn-based SAF structure, with an increasing annealing temperature, the exchange bias field decreases [22,23]. It may be beneficial for the alignment of the pinned layer easy axis along the transverse direction of the patterned elements because the induced anisotropy field is generally smaller than H_ex_. The deviation of the pinning direction depends also on the value of the field applied during the thermomagnetic treatment [18]. In order to further optimize this full bridge sensor, a proper annealing condition needs to be exploited.

## 4. Conclusions

We have successfully designed and fabricated a novel angular GMR sensor in a full Wheatstone bridge by depositing one GMR film stack and utilizing simple patterned processes and a concise post-annealing procedure based on a special layout. The angular GMR sensor is of good linear performance and achieves a sensitivity of 0.112 mV/V/Oe at the annealing temperature of 260 °C in the magnetic field range from −50 Oe to +50 Oe. This work provides a design and method for GMR sensor manufacturing that is easy for implementation and suitable for mass production. The optimization of several aspects and more quantitative analyses are still needed in future work on this sensor.

## Figures and Tables

**Table 1:** The performance of the full bridge-based GMR sensors at different annealing temperatures.

Annealing Temprature	Sensitivity (mV/V/Oe)	Angles of Pinned Layer Magnetization in Arm1	MR Ratio of individual Element
260 °C	0.112	71.7°	6.12%
250 °C	0.093	71.6°	5.25%
240 °C	0.074	80.5°	5.36%
